# Valorization of Byproducts of Hemp Multipurpose Crop: Short Non-Aligned Bast Fibers as a Source of Nanocellulose

**DOI:** 10.3390/molecules26164723

**Published:** 2021-08-04

**Authors:** Sara Dalle Vacche, Vijayaletchumy Karunakaran, Alessia Patrucco, Marina Zoccola, Loreleï Douard, Silvia Ronchetti, Marta Gallo, Aigoul Schreier, Yves Leterrier, Julien Bras, Davide Beneventi, Roberta Bongiovanni

**Affiliations:** 1 Politecnico di Torino, Department of Applied Science and Technology, Corso Duca degli Abruzzi 24, 10129 Turin, Italy; vijayaletchumy13@s.unikl.edu.my (V.K.); silvia.ronchetti@polito.it (S.R.); marta.gallo@polito.it (M.G.); roberta.bongiovanni@polito.it (R.B.); 2 Universiti Kuala Lumpur, Malaysian Institute of Chemical and Bioengineering Technology (UniKL MICET), Lot 1988 Kawasan Perindustrian Bandar Vendor, Taboh Naning, Alor Gajah, Melaka 78000, Malaysia; 3 Consiglio Nazionale delle Ricerche, Istituto di Sistemi e Tecnologie Industriali Intelligenti per il Manifatturiero Avanzato, 13900 Biella, Italy; alessia.patrucco@stiima.cnr.it (A.P.); marina.zoccola@stiima.cnr.it (M.Z.); 4 Université Grenoble Alpes, CNRS, Grenoble INP, LGP2, F-38000 Grenoble, France; Lorelei.Douard@lgp2.grenoble-inp.fr (L.D.); julien.bras@grenoble-inp.fr (J.B.); davide.beneventi@pagora.grenoble-inp.fr (D.B.); 5 Laboratory for Processing of Advanced Composites (LPAC), Ecole Polytechnique Fédérale de Lausanne (EPFL), CH-1015 Lausanne, Switzerland; aigoul.schreier@epfl.ch (A.S.); yves.leterrier@epfl.ch (Y.L.)

**Keywords:** nanocellulose, hemp, waste valorization, nanopaper, lignocellulosic fibers

## Abstract

Nanocellulose was extracted from short bast fibers, from hemp (*Cannabis sativa* L.) plants harvested at seed maturity, non-retted, and mechanically decorticated in a defibering apparatus, giving non-aligned fibers. A chemical pretreatment with NaOH and HCl allowed the removal of most of the non-cellulosic components of the fibers. No bleaching was performed. The chemically pretreated fibers were then refined in a beater and treated with a cellulase enzyme, followed by mechanical defibrillation in an ultrafine friction grinder. The fibers were characterized by microscopy, infrared spectroscopy, thermogravimetric analysis and X-ray diffraction after each step of the process to understand the evolution of their morphology and composition. The obtained nanocellulose suspension was composed of short nanofibrils with widths of 5–12 nm, stacks of nanofibrils with widths of 20–200 nm, and some larger fibers. The crystallinity index was found to increase from 74% for the raw fibers to 80% for the nanocellulose. The nanocellulose retained a yellowish color, indicating the presence of some residual lignin. The properties of the nanopaper prepared with the hemp nanocellulose were similar to those of nanopapers prepared with wood pulp-derived rod-like nanofibrils.

## 1. Introduction

Varieties of *Cannabis sativa* L. with δ-tetrahydrocannabinol (THC) levels below the legal limit are commonly referred to as industrial hemp, or simply hemp. Hemp is an annual crop, suitable for European climates. It is sustainable as it requires a low water and fertilizer input and has no need for herbicides or insect repellants; in addition, it improves soil structure, acting as an excellent break crop in crop rotations, potentially enhancing the yield of other food crops, and can adapt to marginal or contaminated land [1,2]. The Italian region of Piedmont has a long history of hemp cultivation, particularly of the local dioecious Carmagnola variety, a giant variety with a very tall stem and high-quality fibers [3]. Once grown for textile applications, it is now primarily grown for the production of seeds, and for the inflorescences and extraction biomass rich in cannabidiol. The stems remain thus as a byproduct, which has to be exploited to make hemp cultivation truly profitable for farmers [4].

The inner woody core of the stem is composed of short and more lignified fibers, the so-called shives, used mainly as animal bedding, garden mulch or construction material [5]. The core is surrounded by bast fibers, which are of two types: the outer ones, called primary fibers, are thicker, longer and richer in cellulose, while the ones closer to the inner core, the secondary fibers, are thinner, shorter and contain a higher amount of lignin [6]. The primary bast fibers from plants harvested at full flowering are suitable for textile applications or high-performance composites: to this aim, after retting they are separated from the shives, with techniques that allow the collection of long and aligned fiber bundles [7,8]. When the cultivation of hemp is multipurpose, the plant is grown to a higher degree of maturity, leading to a higher lignification of the fibers and to an increased content of secondary bast fibers; the stems are then typically processed with so-called “disordered” decortication methods. The fibers recovered in this way are non-aligned, shorter and of lower quality [1,9], thus being unsuitable for many industries such as textiles, high-performance composites or quality paper [7,10,11].

Bast fibers are multicellular and have a hierarchical structure. They are bundles of single cells called individual fibers, cemented together by the middle lamella, a layer composed primarily of lignin, pectin and small amounts of proteins [12]. Each individual fiber has a hollow central part called the lumen, surrounded by a thick secondary cell wall and a thinner primary cell wall. The cell walls are made of cellulose with a semi-crystalline fibrillary structure, the so-called microfibrils, embedded in an amorphous matrix of hemicellulose, pectin and lignin [13,14,15,16]. Traditionally, crystalline and amorphous cellulose regions are thought to alternate along the microfibrils, while recent studies suggest that amorphous cellulose chains may be located preferentially at the surface of the microfibrils, and the crystalline cellulose chains in the interior [17]. Finally, small amounts of bound water, waxes, pigments and traces of inorganic compounds are also present in the fibers.

Hemp fibers, together with many other fibers from annual plants, have been proposed as a lignocellulosic biomass for the production of nanocellulose, both nanofibrillated and nanocrystalline. Cellulose in hemp fibers has been reported to range from about 55% to 80% depending on the variety and maturity of the plant, and the type of fiber, i.e., primary or secondary bast fibers, or shives [6,18,19,20]; the lignin content is lower than in wood, particularly in the bast fibers, allowing for milder fiber delignification and purification processes [21,22]. Some attempts have therefore been made to use hemp fibers for the production of nanocellulose through chemical, enzymatic and/or mechanical defibrillation methods. Most of the processes either use bleached pulps or include a bleaching step, or involve an oxidative treatment, such as TEMPO (2,2,6,6-tetramethylpiperidine-1-oxyl)-mediated oxidation [23,24,25,26,27,28,29].

Bleaching performed with chlorine or (hypo)chlorite, the latter also being used for TEMPO oxidation, is a common procedure. However, it results in the release of organic halides and other toxic compounds [30]. Elemental chlorine free (ECF) pulp bleaching processes, using chlorine dioxide or alkali H_2_O_2_, are more environmentally acceptable [31], despite concerns related to health and explosion hazards [30,32]. The sustainability of the current H_2_O_2_ production process is also under scrutiny [33]. Greener approaches, avoiding both bleaching and oxidation, have recently been proposed for the preparation of nanocellulose from hemp fibers and shives: the main examples are steam explosion followed by ball milling and sonication [34], dual asymmetrical centrifugation [35], or enzymatic treatment with endoglucanase followed by sonication, combined with a pretreatment with NaOH, ultrasounds and microwaves [36].

In this work, we report the production of nanocellulose from non-aligned hemp bast fibers from a multipurpose crop, to provide added value applications for these abundant and cheap agricultural residues. We used a chemical pretreatment with alkali and acid, adapted from Wang et al. [29], to remove non-cellulosic compounds, followed by an enzymatic pretreatment and finally by mechanical defibrillation with an ultrafine friction grinder. As the raw fibers contained only a very small amount of lignin, in an effort to provide a more environmentally friendly process, we avoided bleaching and other oxidative treatments. The fibers were analyzed after the pretreatment steps; to evaluate the properties of the obtained nanocellulose and assess its performance for possible applications, nanopaper was prepared and characterized in this work.

## 2. Materials and Methods

### 2.1. Materials

Non-aligned bast fibers from hemp (*Cannabis sativa* L.) plants of the Carmagnola variety, harvested at seed maturity, non-retted, and mechanically decorticated in a “disordered line”, were supplied by Assocanapa (Carmagnola, Italy). The fibers were cleaned and disentangled with a Mixicomber machine (O. M. Alvaro Mason & C S.N.C., Valle Mosso, Italy) prior to use.

The chemicals used for the pretreatment were sodium hydroxide pellets (Carlo Erba Reagents S.A.S., Val de Reuil, France) and hydrochloric acid, 37% (Sigma-Aldrich, Inc., St. Louis, MO, USA); ultrapure water from a Millipore Direct-Q 3 UV system (Merck KGaA, Darmstadt, Germany) was used to dilute them to the desired concentrations. For the enzymatic treatment, a buffer was prepared using acetic acid ≥99.7% and sodium acetate trihydrate ≥99.0% (Sigma-Aldrich, Inc., St. Louis, MO, USA); the enzyme was FiberCare^®^ R cellulase 4890 ECU (Novozymes, Copenhagen, Denmark).

### 2.2. Production of Nanocellulose from Hemp Fibers

The disentangled hemp fibers (raw fibers) were chopped using a kitchen grinder, and then underwent a number of chemical and mechanical pretreatments before the final mechanical fibrillation step, as detailed in the following; they were never dried during the procedure. After each pretreatment step, samples of fibers were set aside for analysis.

The chopped hemp fibers were soaked in a 10 wt% aqueous sodium hydroxide (NaOH) solution at room temperature for two hours, with mild agitation, then in a 1M aqueous solution of hydrochloric acid (HCl) for one hour at 80 °C, and finally, in a 2 wt% aqueous NaOH solution for one hour at 80 °C. The ratio of liquid to fibers was equal to 1 L of each solution per 20 g of dry chopped hemp fibers. Between each step, the fibers were rinsed with water, with the aid of a 120-mesh sieve, until the pH was in the 6 to 8 range, and were drained of excess water, but not allowed to dry.

The chemically pretreated fibers were diluted to a 10 wt% concentration in demineralized water and processed with an immersion kitchen blender to obtain a finer pulp. The pulp was then further refined in a PFI mill-type laboratory beater (Metrotec SA, Kirchheim, Germany) at 2500 rpm, prior to the treatment with the FiberCare^®^ cellulase enzyme. The concentration of the enzyme was 300 ECU per gram of dry fibers. The refined fibers, placed in a reactor equipped with an overhead stirrer and a heated water bath, were further diluted to a 2 wt% concentration using a pH 5 acetic acid/sodium acetate trihydrate buffer solution. The temperature was raised to 50 °C, and then the enzymes were added: the fibers were left to stir for 2 h, before raising the temperature to 80 °C for 10 min to deactivate the enzymes. The pulp was then vacuum filtered using a precision woven screening polyamide 6.6 fabric with 1 µm mesh opening (NITEX, Sefar AG, Heiden, Switzerland), and re-dispersed in demineralized water. The filtration procedure was repeated three times in order to reach a neutral pH.

Finally, to obtain the nanocellulose, demineralized water was added to the enzymatically treated pulp, to obtain a solids content of approximately 1.4 g/L. The pulp was defibrillated in a Supermasscolloider ultra-fine friction grinder (Model MKZA6-2, disk model MKG-C 80, Masuko Sangyo Co., Ltd., Kawaguchi, Japan). The clearance gauge was initially set to the 0 position, and the suspension was passed 8 times through the grinder in order to homogenize it. Then, the suspension was passed through the grinder 4 times with the gauge set at −5, 8 times with the gauge set at −10, 1 time with the gauge at −12, and finally 2 times with the gauge at −15. A small sample of the nanocellulose aqueous suspension was dried overnight at 100 °C in order to verify the actual concentration, which was found to be 1.3 wt%.

### 2.3. Production of Nanopaper

Nanopaper handsheets were prepared using the 1.3 wt% nanocellulose aqueous suspension, as obtained after the ultrafine friction grinding, by filtering through a Rapid-Köthen standard sheet former (Frank-PTI, Birkenau, Germany) equipped with a precision woven screening polyamide 6.6 fabric with 1 µm mesh opening (NITEX, Sefar AG). The sheets were then dried in a dryer at 90 °C for 20 min, between two layers of the polyamide 6.6 fabric. The final thickness was 55 ± 1 µm.

### 2.4. Characterization Methods

To measure the insoluble lignin, an acid treatment was applied to the fibers to completely hydrolyze cellulose and hemicellulose. First the fibers were treated with a 72% H_2_SO_4_ solution for 2 h at room temperature, and then the acid solution was diluted to 3% and the temperature was raised to the boiling point for 4 h. Then the suspension was left to decant overnight, and was then filtered to recover the insoluble lignin, which was weighed after drying overnight. To determine the soluble lignin content, 50 mL of the filtrate of the hydrolysis was collected, and the absorbance at 205 nm was measured with a UV-Vis spectrophotometer, using a 3% H_2_SO_4_ solution as blank.

The yield, expressed as a percentage, after the chemical pretreatment and after the entire process was calculated as yield (%) = 100 *M_i_/M_chop_*, where *M_chop_* was the mass of chopped fibers, and *M_i_* was the mass of fibers after the selected step, i.e., after the chemical pretreatment, and after the ultrafine friction grinding.

Samples of the hemp fibers taken after each pretreatment step were examined in reflection mode with a BX53M optical microscope (Olympus Italia S.R.L., Segrate MI, Italy) and in transmission mode with an Axio Imager M1 optical microscope equipped with an AxioCam MRc 5 digital camera (Carl Zeiss, Jena, Germany). For the observation, the fibers were dispersed at low concentration in distilled water, and a small amount was poured onto a glass slide and covered with a glass lid. Images were taken at various magnifications.

Samples were also observed with a field emission scanning electron microscope (Supra 40 FE-SEM, Carl Zeiss). The pretreated fibers were dried in an oven at 60 °C and spread on carbon tape. The nanocellulose suspension was further diluted with addition of distilled water, and a droplet was poured onto a transmission electron microscope (TEM) grid and left to evaporate at room temperature. The specimens were coated with Cr.

In order to obtain transmission electron microscope (TEM) images of the nanosized particles, a small sample of the nanocellulose suspension was diluted and decanted to separate the larger fibers. Droplets of the decanted nanocellulose suspension were deposited onto glow-discharged carbon-coated TEM grids. After a few minutes, the excess liquid was blotted with filter paper and the preparation was negatively stained with 2 wt% uranyl acetate. The excess stain was blotted and the specimen allowed to dry. The specimens were observed with a JEOL JEM-2100-Plus microscope operating at 200 kV, and images were recorded with a Gatan Rio 16 digital camera.

The dimensions (length and width) of the fibers after each pretreatment step were assessed from the optical microscope, FESEM and TEM images by means of the software ImageJ (release 1.52 s, Wayne Rasband, National Institutes of Health, 9000 Rockville Pike, Bethesda, MD, USA).

Fourier transform infrared (FT-IR) analysis was performed with a Nicolet iS50 spectrometer (Thermo Fisher Scientific Inc., Waltham, MA, USA), equipped with a Smart iTX-Diamond attenuated total reflectance (ATR) accessory, in the 4000–550 cm^−1^ range with 32 scans per spectrum and a 4 cm^−1^ resolution. To obtain a flat surface for analysis, the samples were poured into a glass Petri dish and dried at 60 °C until constant weight was reached. These samples were then directly put on the FTIR diamond for analysis.

Thermogravimetric analysis (TGA) was performed from 25 °C to 800 °C with a heating rate of 20 °C min^−1^, under a 60 mL min^−1^ N_2_ flux, to prevent thermo-oxidative processes (TGA/SDTA 851e, Mettler-Toledo (Schweiz) GmbH, 8606 Greifensee, Switzerland). Prior to analysis, all samples were dried in an oven at 100 °C for 1 h.

For differential scanning calorimetry (DSC), for each sample two heating cycles were performed at temperatures between −70 °C and 250 °C at a heating rate of 10 °C min^−1^ under N_2_ flow (DSC 1 STARe System, Mettler Toledo).

X-ray diffraction characterization was performed on samples of dry fibers and nanocellulose using a Panalytical X’Pert PRO (Cu Kα radiation) diffractometer, with a PIXcel detector, a solid-state detector with rapid readout time and high dynamic range. Data collection was performed at 40 kV and 40 mA, between 10° and 40° 2θ, with a step of 0.02° 2θ and a wavelength of 1.54187 Å. To allow rapid comparison of fiber samples, the crystallinity index (CI) was calculated according to Segal’s equation [37]:

(1) $$CI=100\times \left(I_{200}-I_{am}\right)/I_{200}$$

where *I*_200_ is the height of the 200 peak and *I_am_* the height of the minimum diffraction intensity between the 200 and the 110 peaks. This method is useful for comparing the relative differences between samples [38]. Crystallite size was determined according to Scherrer’s equation [39] by comparing the profile width of a standard profile with the sample profile. Scherrer’s equation relates the width of a powder diffraction peak to the average dimensions of crystallites in a polycrystalline powder:

(2) $$D=K\lambda /\beta_{\left(2\theta \right)hkl}cos\theta$$

where *β* is the crystallite size contribution to the peak width at half maximum (FWHM) in radians, *K* (shape factor) is a constant whose value is close to unity, and *D* is the average thickness of the crystal in a direction normal to the diffracting plane *hkl*. Profile fits were performed using X′Pert High Score Plus, using pseudo-Voigt peak function with Kα_1_ and Kα_2_ fitting on a background stripped pattern. The sample-induced peak broadening *β* was determined by subtracting the instrumental peak width from the measured peak width. The instrumental broadening was determined using LaB_6_ powder (NIST SRM^®^660a, size of crystallites in the 2–5 µm range).

In order to assess the compatibility of the hemp nanocellulose with different solvents, demineralized water, ethanol or acetone was added to the nanocellulose suspension, to obtain a 0.25 wt% nanocellulose concentration. The suspensions were then homogenized with a T10 Ultra Turrax and put into closed vials; their settling was observed for 12 days.

Dynamic mechanical analysis (DMA) was performed in tensile mode on rectangular specimens of nanopaper handsheet. The temperature was increased from −100 °C to 180 °C at a 3 °C min^−1^ rate, the frequency was set at 1 Hz, and the strain was set at 0.01% (TTDMA, Triton Technology Ltd., Keyworth, UK). The specimens had a length of 10 mm between the clamps, and a width of 6 to 8 mm.

Tensile tests were performed on rectangular specimens cut from the handsheet, using a Dual Leadscrew 5KN tensile testing stage (Deben UK Ltd., Suffolk, UK), with a speed of 1.0 mm/min.

To measure the oxygen transmission rate (OTR) and the water vapor transmission rate (WVTR), an oxygen permeation analyzer Systech 8001 and a water vapor permeation analyzer Systech 7001 (both by Systech Illinois, Thame, UK), were used, respectively. In the two parallel chambers of each instrument, specimens of the nanopaper were mounted using a steel mask with a circular opening of 5 cm^2^. During the conditioning cycle, dry nitrogen was used to purge the chambers. Then for the measurements, a flow of either pure oxygen gas (for OTR) or wet nitrogen gas (for WVTR) was fluxed to one side of the film. The OTR measurement was performed at 23 °C and 50% RH, while the WVTR measurement was performed at 38 °C and 50% RH. The results were collected when the oxygen or water vapor transmission rates reached a steady state. In the text that follows, all the reported OTR values are expressed in cm^3^ m^−2^ day^−1^ bar^−1^ and the oxygen permeability (P_O2_) values in cm^3^ mm m^−2^ day^−1^ bar^−1^, while the reported WVTR values are expressed in g m^−2^ day^−1^ and the water vapor permeability (P_H2O_) values in g mm m^−2^ day^−1^ atm^−1^: these units will be omitted to improve readability.

## 3. Results and Discussion

The composition of Carmagnola hemp bast fibers collected after dry mechanical treatment of the stalks was reported to be approximately 61–69% cellulose, 14–17% hemicellulose and 3–5% lignin [40]. Similarly, the raw fibers used in this work contained a low amount of lignin, i.e., 3.9% insoluble lignin and 0.7% soluble lignin.

**Figure 1:**
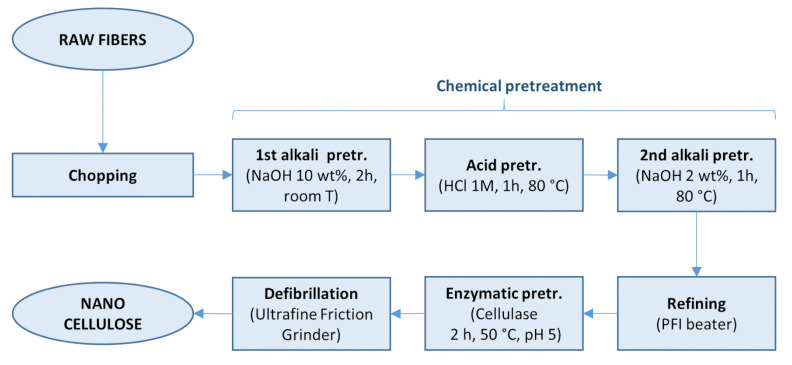
Scheme of the process for obtaining hemp nanocellulose.

The process for obtaining the nanocellulose, detailed in the experimental section, is summarized in Figure 1: the fibers were chopped, and then underwent a number of pretreatments with alkali, acid and a cellulase enzyme, before the final mechanical defibrillation step. Characterization was performed on the hemp fibers after each pretreatment step, in order to elucidate the effect of each pretreatment on the fiber structure and on the final nanocellulose. The yield after the chemical pretreatment was about 60%, which accounts for the removal of non-cellulosic compounds, losses of smaller fibers through the sieve during the rinsing steps, and the removal of adsorbed water in the raw fibers upon drying. This result is compatible with the cellulosic content reported in the literature for Carmagnola hemp fibers. At the end of the treatment, the nanocellulose suspension recovered after the mechanical defibrillation accounted for 97% of the weight of the chemically treated fibers, therefore resulting in a global yield of 57%. The nanocellulose suspension, as obtained after mechanical defibrillation, was used to prepare nanopaper, which was then characterized.

### 3.1. Evolution of the Dimensions and Morphology of the Fibers during the Pretreatment

**Figure 2:**
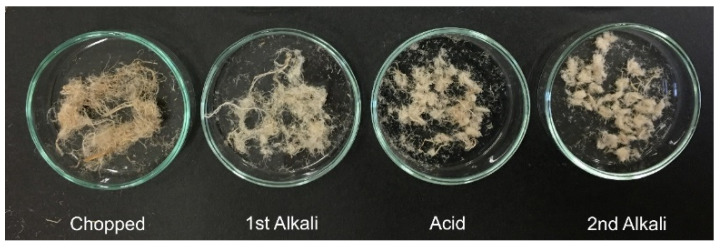
Fiber samples showing the color change after different steps of the chemical pretreatment.

A photo of the hemp fibers, chopped and after each step of the chemical pretreatment, showing the color change of the fibers, is shown in Figure 2.

After the first alkali pretreatment, with a 10 wt% NaOH solution for 2 h at room temperature, the fibers had a lighter color than the chopped ones, due to the removal of the non-cellulosic compounds of the cell wall, i.e., hemicellulose and lignin. Accordingly, the alkali solution recovered after the treatment had a dark yellow color. After the acid and the second alkali pretreatment, with 1M HCl and with 2 wt% NaOH, respectively, both performed for 1 h at 80 °C, the fibers still retained a yellowish color. As the corresponding solutions recovered at the end of these treatments also had a pale yellow color, it is inferred that some hemicellulose and lignin that were protected inside the fibers were however further removed during these steps.

**Figure 3:**
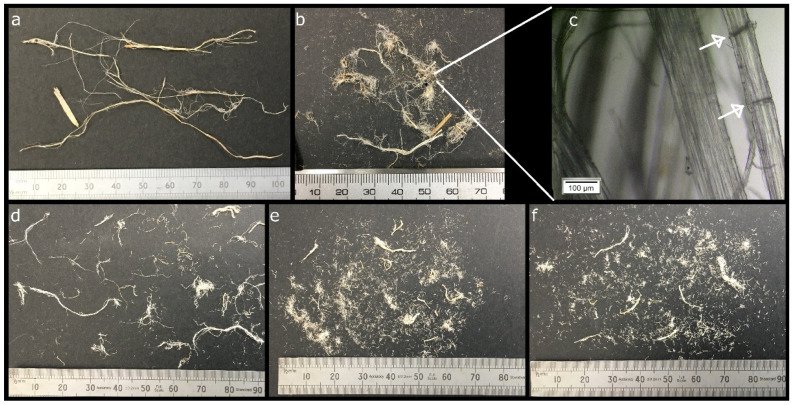
Images showing the evolution of the dimensions of the fibers: (**a**) raw fibers; (**b**) chopped fibers; (**c**) optical micrograph of chopped fibers in which dislocations appear as transversal dark lines, indicated by the arrows; (**d**) fibers after the first alkali treatment; (**e**) fibers after the acid treatment; (**f**) fibers after the second alkali treatment.

**Figure 4:**
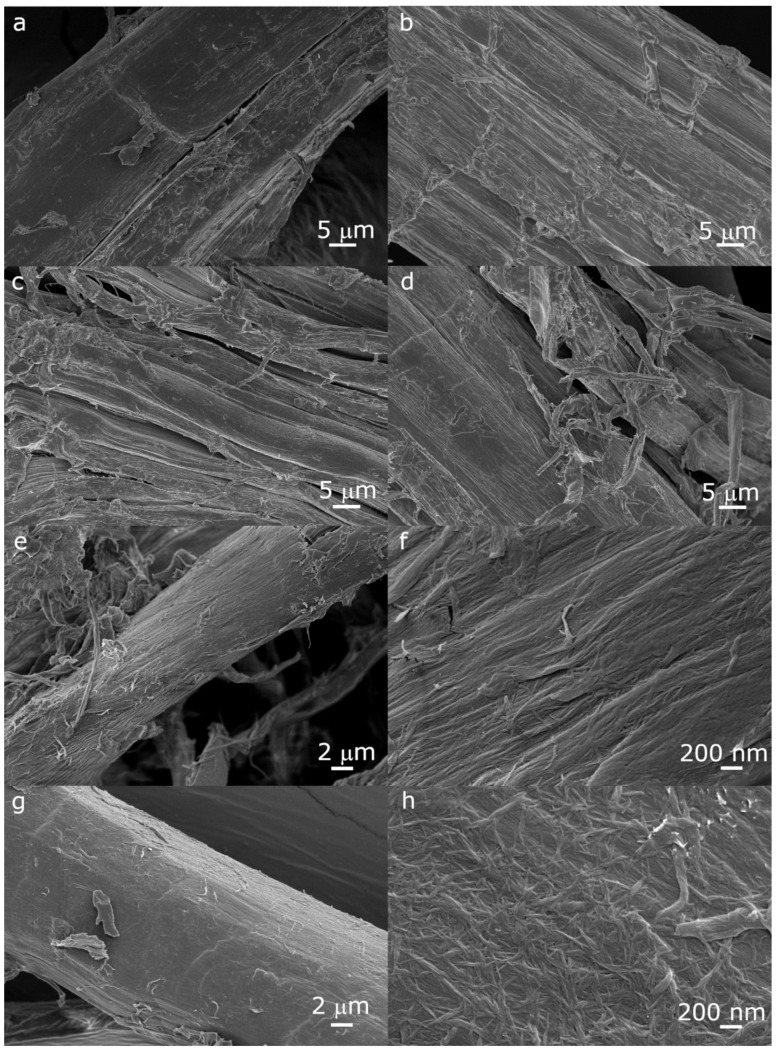
FESEM images of the fibers after each pretreatment step, showing the appearance of their surface: (**a**) chopped fibers; (**b**) after the first alkali treatment; (**c**) after the acid treatment; (**d**) after the second alkali treatment; (**e**,**f**) after refining; (**g**,**h**) after the enzymatic treatment. Images (**f**,**h**) show that the microfibrils start appearing at the surface of the fibers after refining and enzymatic pretreatment.

The evolution of the dimensions of the fibers during the chemical pretreatment is shown in Figure 3, and Figure 4 shows FESEM images of the surface of the fibers after each pretreatment step. Additional optical microscopy and FESEM images can be found in the open access Zenodo repository [41].

The raw hemp fiber sample was composed of primary and secondary bast fibers with a wide range of dimensions, with widths of few micrometers to several hundred micrometers and lengths up to 150 mm, and a small number of fragments of shives, with widths of up to 2 mm and lengths up to 10–20 mm.

After chopping, the maximum fiber length was reduced to about 40 mm, with a large amount of shorter fibers, so that the median length was about 5 mm; dislocations (defects), appearing as transversal dark lines in the optical micrographs, could be observed. The mechanical action of the mixer blades not only cut the fibers, but also favored the splitting of the bundles of individual fibers. The fiber bundles had sections of irregular shapes, while the individual fibers had more round sections. Fiber widths were mostly in the 1–500 µm range, with a large part of the fibers with widths below 80 µm. Some fragments of shives with widths up to about 1 mm were still visible. The surface of the fiber bundles was rough as the middle lamella still surrounded the individual fibers; a few thin fibers with micron to submicron dimensions departed from the surface of the fiber bundles (Figure 4a).

After the first alkali pretreatment, the fibers still had lengths up to 40 mm, although, with respect to the chopped fibers, the number of shorter fibers increased, with the median value for the length decreasing to 2 mm. The widths of the fibers were similar to those of the chopped fibers. Due to the removal of the middle lamella, the individual fibers composing the bundles became clearly visible, but did not separate much further (Figure 4b), as even high concentrations of NaOH have been reported to not be able to completely remove all the lignin and pectin cementing the bundles [42]. The estimated widths of the individual fibers, 3 μm to 20 μm, were consistent with those reported in the literature [42,43].

The acid pretreatment overall reduced the length of the fibers, likely due to acid hydrolysis, which is known to weaken the fibers at dislocations [44]. The observed lengths were in the 0.1 mm to 16 mm range, with a median length of 0.9 mm. This range of lengths is in line with that reported for hemp individual fibers [43]. The treatment favored the separation of the individual fibers (Figure 4c), as also indicated in other works [23]. As a result, the amount of finer fibers increased, with the median value for fiber width decreasing to about 16 μm, although the upper limit for the observed fiber widths did not change, as not all the bundles were disrupted.

The second alkali pretreatment further loosened the individual fibers, removing non-cellulosic compounds; some thin ribbon-like filaments, probably parts of damaged individual fibers, could be seen protruding from their surfaces (Figure 4d). A slightly higher amount of shorter fibers could be observed, while the observed widths did not substantially change with respect to the previous step.

Refining greatly reduced the length and width of the fibers. The fiber length was in the 400 μm to 10 μm range, with a median value of about 70 μm. The majority of the fibers had widths ranging from submicron to about 20 μm, and the median value was close to 3 μm, and on the surface of the individual fibers, a fibrous structure of nanometric fibrils appeared (Figure 4e,f).

The enzymatic pretreatment did not further significantly change the fiber dimensions; nevertheless, the surface of the fibers was “cleaner”, and a structure of fibrils of nanometric dimensions became well visible (Figure 4g,h).

**Figure 5:**
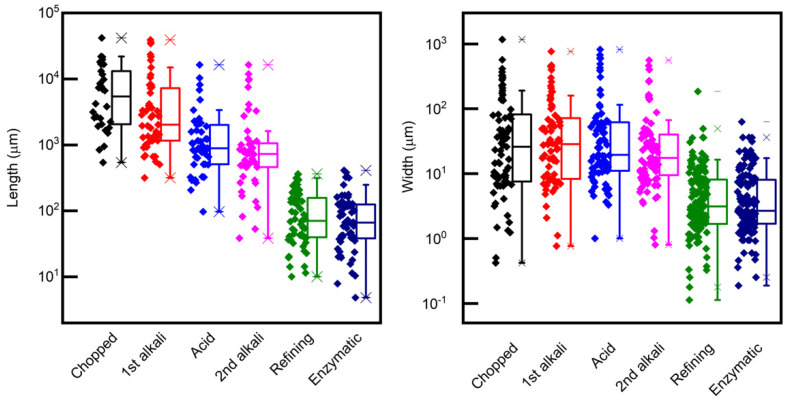
Boxplots of the lengths and widths of the hemp fibers after each pretreatment step. The measured data points are shown close to the corresponding boxes.

To summarize, the dimensions of the hemp fibers after each pretreatment step are compared in the box plots of Figure 5. One can conclude that a reduction in the length was obtained gradually throughout the pretreatments, with an abrupt decrease after refining. On the other hand, the width of the fibers did not decrease significantly with the chemical pretreatments, while a significant decrease was obtained with mechanical refining, suggesting that mechanical energy input is needed to separate the individual fibers. The pretreatment with enzymes did not significantly further reduce the fiber lengths and widths.

### 3.2. Morphology and Dimensions of the Nanocellulose

**Figure 6:**
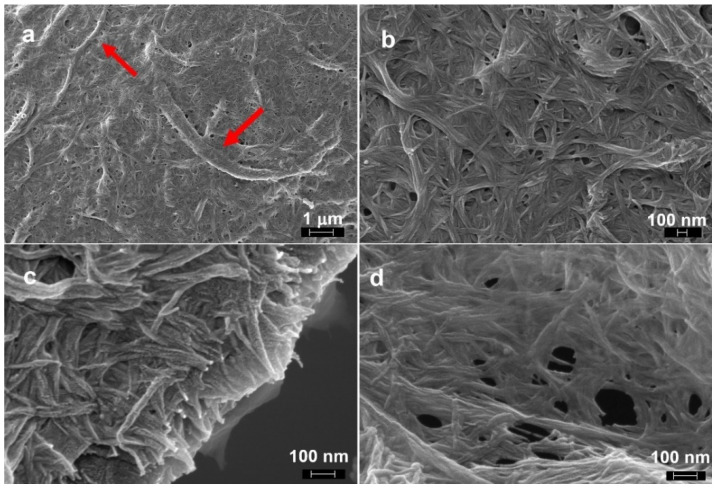
FESEM images of the nanocellulose, composed of nanosized nanofibrils mixed with micron sized fibrils: (**a**) lower magnification image in which some micron sized fibrils are indicated by arrows and (**b**–**d**) higher magnification images showing the network of nanosized fibrils.

**Figure 7:**
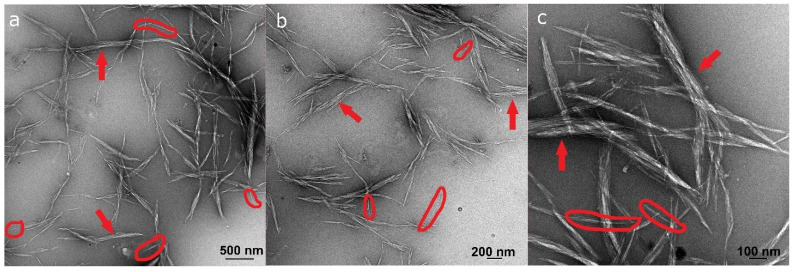
TEM images of the nanosized fraction of the nanocellulose suspension at (**a**) low magnification, (**b**) intermediate magnification and (**c**) high magnification. In all images, examples of individual fibrils are circled and examples of stacks of fibrils are indicated by arrows.

After the series of pretreatments, the fibers, still of micrometric dimensions, were subjected to mechanical defibrillation in an ultra-fine friction grinder, to obtain nanocellulose. The FESEM observation of the nanocellulose suspension highlighted that nanosized fibrils were present together with larger micron sized fibers (Figure 6). The nanosized fraction, separated by decanting as mentioned in Section 2.4 for the inspection by TEM (Figure 7), was composed of individual nanofibrils with lengths of 100–300 nm and widths of 5–12 nm, and stacks of fibrils with lengths of 380 nm to 3 μm and widths between 20 nm and 200 nm. The dimensions of the individual nanofibrils were comparable to those of the cellulose nanocrystals (CNC) obtained by Luzi et al. from Carmagnola hemp bast fibers by acid hydrolysis and to those obtained by Xu et al. from hemp and flax bast fibers by endoglucanase treatment (Xu et al., 2013; Luzi et al., 2014).

### 3.3. FTIR Characterization

**Figure 8:**
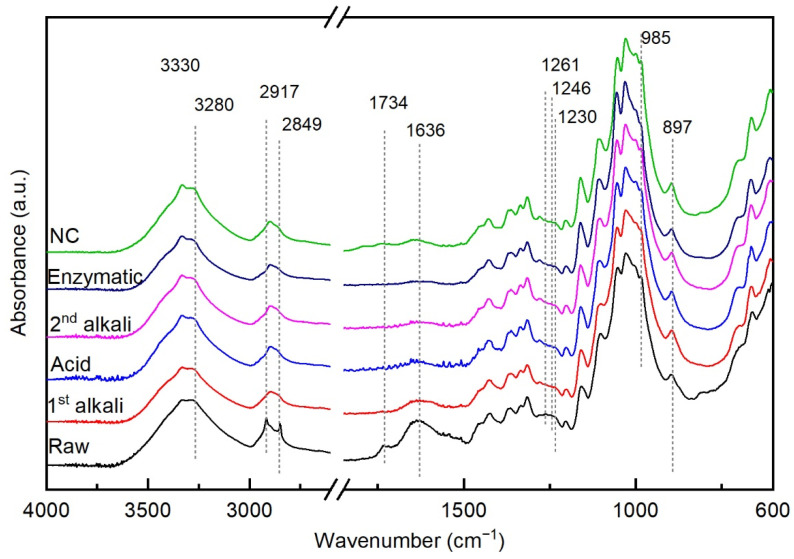
FTIR spectra of the hemp fibers, raw and after the pretreatments, and of the hemp nanocellulose.

The FTIR spectra of the raw hemp fibers and of the fibers after each treatment are shown in Figure 8. In the spectrum of the raw fibers, absorption bands that correspond to the stretching and bending vibrations of the main compounds present in lignocellulosic fibers, such as cellulose, hemicellulose and lignin, were identified. The peak assignments are reported in Table 1.

The first alkali pretreatment caused the disappearance of the two sharp peaks at 2917 cm^−1^ and 2849 cm^−1^, indicating the removal of fatty acids. Furthermore, the peaks at 1734 cm^−1^ and 1246 cm^−1^, as well as at 1261 cm^−1^ and 1230 cm^−1^ decreased, confirming that the content of hemicellulose and lignin, respectively, diminished. The peak at 1636 cm^−1^ assigned to bound water strongly decreased. Peaks at ca. 1104 cm^−1^, 1053 cm^−1^ and 1029 cm^−1^, which can be assigned to cellulose alcohols, became sharper. The broad band between 3700 cm^−1^ and 3000 cm^−1^ showed some changes, particularly in the intensities at 3330 cm^−1^ and 3280 cm^−1^, which may indicate changes in the type of hydrogen bonds formed within and between the cellulose fibrils.

The following acid and second alkali pretreatments caused a further slight change in the spectra following the same trends, indicating the removal of some residual non-cellulosic compounds, in agreement with the visual observation reported in Section 3.1. After the enzymatic pretreatment and after the defibrillation in the grinder, only some changes in the relative heights of the peaks in the region from 1104 cm^−1^ to 985 cm^−1^ were detected, possibly indicating changes in the conformation of the hydroxyl groups of cellulose. The enzyme-pretreated fibers showed a lower intensity of the peak at 3280 cm^−1^ in comparison to that of the peak at 3330 cm^−1^, suggesting the disruption of intramolecular hydrogen bonds, as well as a lower intensity of the bands at 1002 cm^−1^ and 985 cm^−1^, compared with the neighboring peaks at 1056 cm^−1^ and 1030 cm^−1^. The spectrum of the dry nanocellulose on the contrary showed an increased intensity of the peaks at 3280 cm^−1^ and at 1002 cm^−1^ and 985 cm^−1^; the increase in the peak at 3280 cm^−1^ suggests that more intermolecular hydrogen bonds were formed upon drying.

### 3.4. X-ray Diffraction

**Figure 9:**
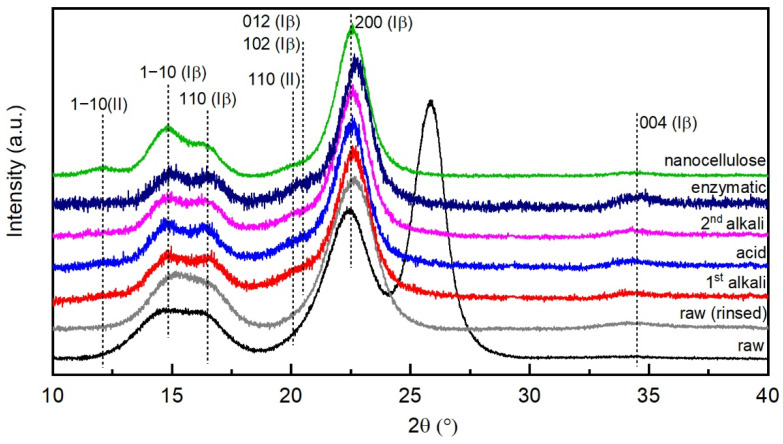
X-ray diffractograms of the hemp fibers at different stages of the process.

Native cellulose typically consists of two crystalline forms, Iα and Iβ, which can coexist in a single microfibril in various proportions depending on the cellulose source. Cellulose Iα, which is predominant in, e.g., algae and bacterial cellulose, has a triclinic unit cell, containing one cellulose chain. Cellulose Iβ, which is the main component in fibers from higher plants such as hemp, cotton and the like, has a monoclinic unit cell, containing two parallel cellulose chains [55,56,57]. All fiber samples in Figure 9 clearly showed diffraction peaks at 2θ values of 14.8°, 16.5°, 22.5° and 34.5°, corresponding to the (1$\bar{1}0$), (110), (200) and (004) crystallographic planes of semi-crystalline cellulose Iβ. Only the raw hemp showed an additional peak at 2θ of 25.9°, which could not be attributed to any of the constituents of natural fibers. As the peak disappeared after rinsing the fibers in cold distilled water, it was attributed to the presence of impurities. Cellulose II exhibits characteristic peaks for the (1$\bar{1}0$), (110) and (020) reflections located at 12.2°, 20° and 22.1°, respectively. While the shoulder around 20° that appeared in the diffractograms of all fiber samples after the first alkali pretreatment may correspond to the superposition of a weak (110) reflection of cellulose II and of (012) and (102) reflections of cellulose Iβ appearing in randomly oriented samples [55,58], a very weak peak at 12.2° was only visible in the nanocellulose pattern. This suggests that only a negligible presence of cellulose II was present in the samples after the alkali treatments. Strong alkali treatments are known to induce a partial transformation of cellulose I to the more stable cellulose II structure, the so-called mercerization. This happens particularly in combination with bleaching treatments [23,25,59,60]. On the other hand, the presence of residual hemicellulose and lignin, which in our case is suggested by FTIR (Section 3.3) and thermogravimetric analysis results (Section 3.5), is thought to inhibit the transformation, possibly by hindering the penetration of alkali in the cellulose fibrils [25].

To estimate the size of the crystallites, we assumed that the lateral crystallite size is the primary contributor to broadening of X-ray peaks. The crystallite size was determined from the broadening of the (200) reflection because it was the most clearly resolved of the three X-ray reflections. The dimension which is measured is in a direction normal to the surface of a crystallite. Table 2 summarizes the positions of the fitted peak and the width of the peak at half height (β), the degree of crystallinity (CI) and the size of the crystallites.

The degree of crystallinity increased abruptly after the first alkali treatment, most probably due to the removal of amorphous materials such as lignin, hemicellulose and waxes. At the same time, the crystallite size increased as well. Then crystallinity slightly increased up to 80% for the final nanocellulose. The progressive decrease in the width at half height values (β) following the chemical pretreatments is reflected in the increase in crystallite sizes. A correlation between crystallite sizes and CI was found, in agreement with other researchers who observed an inverse correlation between X-ray broadening (β) and X-ray fractional crystallinity [61,62,63]. This is consistent with the recent theory that amorphous cellulose concentrates at the surface of crystallites [17]. Smaller crystallites have a larger surface area to volume ratio, and therefore a larger fraction of amorphous cellulose surrounding the crystalline part, leading to lower CI, compared with larger crystallites, which instead have smaller surface area to volume ratios, and therefore lower amounts of amorphous cellulose surrounding the crystalline fraction, resulting in higher CI.

### 3.5. Thermal Analysis

**Figure 10:**
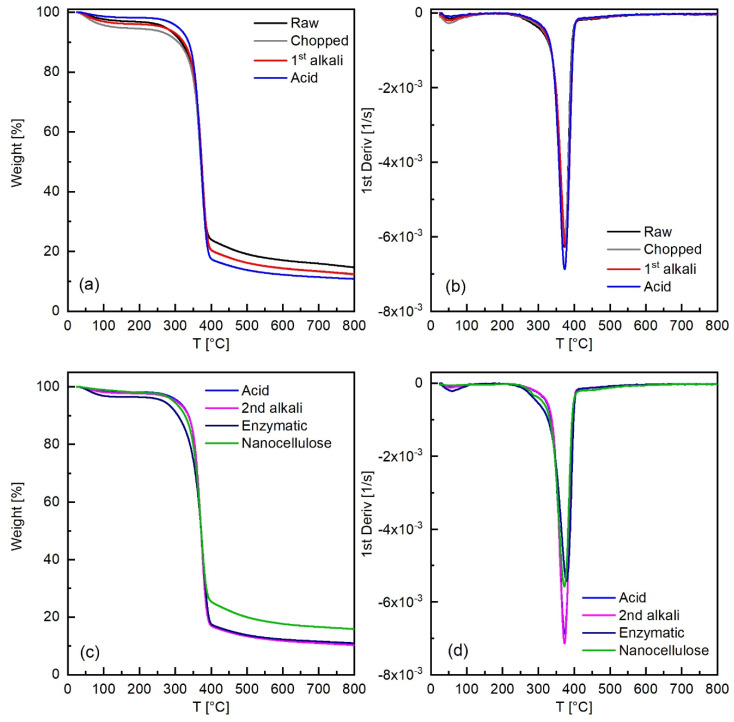
Thermogravimetric analysis under N_2_ of the fibers, as received and after different pretreatment steps: (**a**,**c**) evolution of the weight as a function of temperature, and (**b**,**d**) first derivative of the weight curve.

The results of the thermogravimetric analysis performed on the raw and pretreated hemp fibers and on the nanocellulose are reported in Figure 10. Generally, all the thermogravimetric curves presented the following features: (i) an initial weight loss due to evaporation of residual water, around 100 °C; (ii) between 250 and 300 °C, the thermal decomposition of hemicellulose; (iii) in the 350–400 °C region, the thermal decomposition of cellulose; (iv) from 250 to 600 °C, the thermal decomposition of lignin, which usually happens in a wide range of temperatures, due to the complexity of the lignin structure; (v) a residue at 800 °C of 11–17 wt%.

Comparing in detail the first derivative of the weight curve of the raw and chopped hemp fibers with that of fibers treated with 10 wt% NaOH, one notices in the latter only a very slight decrease in the two shoulders, corresponding to thermal decomposition of hemicellulose and lignin, which appeared at the two sides of the peak attributed to thermal decomposition of cellulose; in addition, the temperature of maximum decomposition rate (T_max_) for cellulose slightly increased after the alkali pretreatment. These small changes can be attributed to the removal, after the 10 wt% NaOH pretreatment, of the non-cellulosic compounds, hemicellulose and lignin, at the surface of the fibers. After the acid pretreatment, with 1M HCl, the low temperature weight loss, associated with the residual water present in the fibers, decreased to less than 2%, consistently with the decrease in the corresponding peak in FTIR analysis. The T_max_ for cellulose increased, while the residual weight at 800 °C decreased to about 11%. These effects are attributed mostly to acid hydrolysis of hemicellulose and cellulose, leading to removal of hemicellulose and to molecular weight decrease for cellulose [64]. The second alkali pretreatment, consistently with what was seen by FTIR analysis, caused only minor changes, in the same direction as the acid pretreatment. After the enzymatic pretreatment, the weight loss at low temperature attributed to residual water increased, and T_max_ decreased: finer fibers are likely to form quite densely packed structures, hindering water removal during the predrying in the oven and slowing down thermal decomposition. The onset of cellulose thermal decomposition was at a lower temperature, probably due to the reduction in the molecular weight caused by the endoglucanase enzyme action.

The main differences in the thermal decomposition behavior of the dry nanocellulose with respect to that of the enzymatically treated sample are an initial slow and almost linear weight loss (2–3%) until about 190 °C, which can be attributed to residual non-freezing water, and the increase both in the onset temperature for cellulose thermal decomposition and in the residual weight at 800 °C, due to the formation of a denser structure upon predrying in the oven.

The DSC thermogram of the dry nanocellulose (Figure S1) during the first heating run showed a broad endotherm peak between 0 °C and 200 °C, centered around 90–95 °C, which was not present in the second heating run. The presence of this peak, attributed to the evaporation of non-freezing water, is consistent with what seen in thermogravimetric analysis. No other transitions appeared in the observed temperature range.

### 3.6. Compatibility of Nanocellulose with Solvents

**Figure 11:**
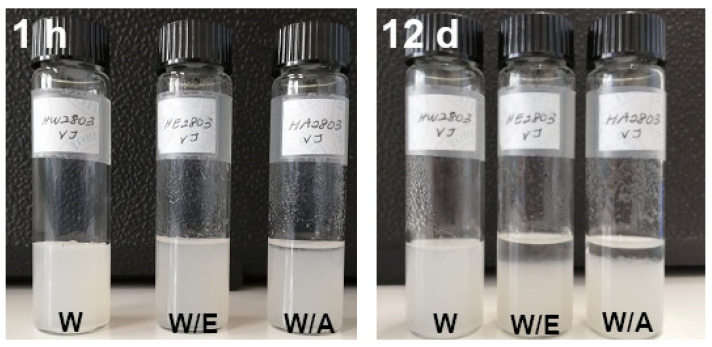
Hemp nanocellulose after settling for 1 h (1 h) and 12 days (12 d) in different solvents (W = water, W/E = water/ethanol, W/A = water/acetone).

As nanocellulose is an efficient reinforcing agent in polymeric composites, where the matrix usually has a low polarity, the compatibility with different solvents was tested. Samples of the suspension collected after the ultrafine friction grinding were diluted by adding either distilled water, ethanol or acetone, to obtain a solids content of 0.25 wt%. The obtained suspensions were left to stand at room temperature for 12 days, as shown in Figure 11. The suspension in water was stable, showing no settling. In water/ethanol and water/acetone, there was some settling of the cellulose fibrils; while after 1 h only the water/acetone solution showed some settling, after 12 days a large settling was visible both in water/ethanol and in water/acetone. The content of ethanol and acetone in the solutions was close to 82 wt%, and the density of the solutions was estimated to be close to 840 kg/m^3^ [65,66]. The settling in the organic solvent mixtures may be attributed to the lower density with respect to water, which allows a higher settling velocity; destabilization of the fibrils in the presence of the organic solvents may also be ascribed to their different polar/dispersive characters.

### 3.7. Use of Nanocellulose for the Preparation of Nanopapers and Their Characterization

**Figure 12:**
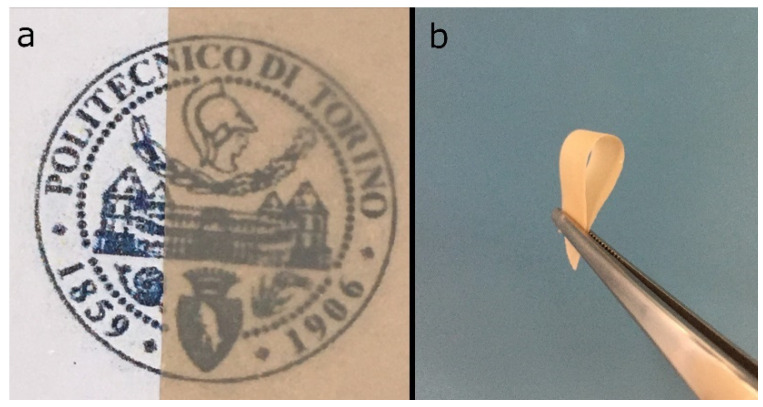
Photos of samples of the hemp MFC handsheet showing (**a**) color and transparency, and (**b**) flexibility.

The nanocellulose was used to prepare nanopapers by a standard procedure based on filtering the suspensions through a Rapid-Köthen standard sheet former and drying. The handsheet obtained from the hemp nanocellulose was transparent with a brownish color and relatively flexible. Photos of samples cut from the handsheet are shown in Figure 12.

**Figure 13:**
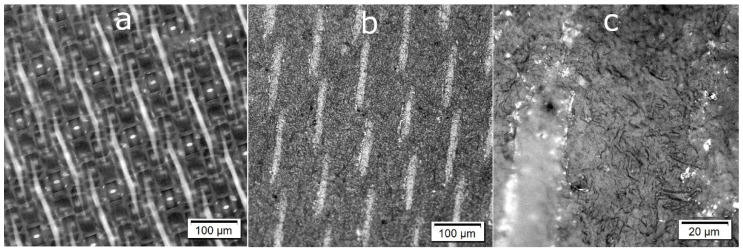
Optical micrographs of (**a**) the surface of the polyamide 6.6 fabric used as a filter in the handsheet formation process and (**b**,**c**) the surface of the handsheet at different magnifications, showing (**b**) the imprinted pattern from the nylon filter and (**c**) the microstructure formed by the nanocellulose.

Figure 13 shows optical micrographs of the surface of the handsheet and of the polyamide 6.6 fabric used in the handsheet formation process. The optical microscopy observation at lower magnification revealed the presence of a larger scale structure on the surface of the nanopaper, repeating the pattern of the PA 6.6 fabric onto which the handsheet was prepared (Figure 13a,b). At a higher magnification, the microstructure of the cellulose fibrils appeared (Figure 13c). Observation with a confocal microscope allowed a 3D reconstruction of the sample surface (see Figure S2).

The results of the DSC and TGA analyses of the handsheet did not show any relevant difference with those reported for the dry nanocellulose (see Figures S1 and S3).

The dynamic mechanical analysis performed on strips cut from the handsheet (Figure S4) showed a storage modulus close to 5 GPa between −100 °C and 180 °C, and did not highlight any phase transition in the entire temperature range. The tensile tests (Figure S5) revealed an elastic behavior, and the rupture was brittle. The Young’s modulus was of the order of 9 ± 2 GPa, in agreement with the results reported for films obtained from cellulose nanocrystals ranging from 3 to 30 GPa [67]. Elongation at break was in the 0.15–0.35% range, and tensile strength was 20 ± 5 MPa, which is lower than literature data ranging from 30 to 130 MPa. The difference can be ascribed to defects in the specimens, in particular small flaws appearing on the sides upon cutting and visible to the naked eye: in fact, the material was very brittle.

Finally, in view of assessing the potential of this material for packaging applications, a preliminary assessment of the barrier properties of the 55 µm thick handsheet was performed. The water vapor transmission rate (WVTR) at 38 °C and 50% RH was 56 ± 8 (i.e., P_WV_ = 94 ± 13). OTR at 23 °C and 50% RH was 3 ± 2 (i.e., P_O2_ = 0.2 ± 0.1). These results are in line with those reported by [68] for nanopapers produced with rod-like nanofibrils from wood sources, with OTR values between 162 and 218, and WVTR values between 2.6 and 16.1, for a thickness of 25 µm.

## 4. Conclusions

We have proven that nanocellulose can be obtained from scrap bast fibers, a byproduct of hemp crops harvested for seed, separated from the shives with a disordered mechanical method. The process included mechanical and chemical treatments but avoided bleaching or oxidation. Thus, it is suggested that these steps, often involving environmentally hazardous chemicals, may not be necessary, unless the color of the fibers has specific application requirements.

It was possible to use the unbleached nanocellulose to fabricate nanopapers: the handsheets were transparent and showed a brownish color. They had thermal, mechanical and permeability properties in line with similar cellulosic materials obtained from non-waste biomass, such as fibers from wood sources.

Thus, hemp is a good source of valuable nanomaterials: the nanocellulose obtained from it is not only suitable for making nanopaper as shown here, but also for nanocomposites. These have in fact been successfully prepared, as described in a separate manuscript [69].

## Figures and Tables

**Table 1:** Main FTIR vibrations in cellulose, hemicellulose and lignin.

Wavenumber (cm^−1^)	Vibration
3700–3000	O–H stretching of hydroxyl groups in polysaccharides (cellulose and hemicellulose) [45]
3330	Valence vibration of hydrogen bonded O–H [46]
3280	O6-H6⋯O3 intermolecular hydrogen bond in cellulose Iβ [47,48,49]
2917, 2849	C–H stretching of aliphatic methylene groups of fatty acids [21,50]
2895	C–H symmetrical stretching of hydrocarbon constituents in polysaccharides [51]
1734	C=O unconjugated stretching of acetyl groups in hemicelluloses [23]
1636	O–H bending/deformation in water bound to cellulose [23]
1595, 1505	Skeletal vibrations in phenolic ring in lignin [46,47]
1462	δCH_2_ asymmetric bending in cellulose I, δCH_3_ asymmetric bending in lignin and hemicelluloses [47]
1424, 1420	δCH_2_ symmetric bending in crystalline cellulose I (strong), in amorphous cellulose (weak) [47,51]
1370	δC–H and δ_s_CH_3_ in cellulose and hemicelluloses [47]
1337	O–H in plane bending in amorphous cellulose [51]
1320	CH_2_ wagging in crystalline [47,51]
1261	C–O stretching, guaiacyl rings in lignin [46,52,53]
1246	stretching vibration of C=O in xylan [54]
1230	C–O stretching of syringyl rings in lignin [52]
1104	C–O vibrations from cellulose secondary alcohols [45]
1051	C–O stretching in hemicellulose, lignin and cellulose secondary alcohols [23]
1034	C–O–C skeletal vibration of polysaccharides ring [47]
1027	C–C, C–OH, C–H ring and side group vibrations in hemicellulose, pectin and cellulose primary alcohols [51]
1002	C–O vibrations from cellulose primary alcohols [45]
985	C–O bending of cellulose alcohols involved in hydrogen bonds [45]
897	Glycosidic bonds symmetric ring-stretching mode of amorphous regions in cellulose [51]

**Table 2:** Values of 2θ_max_ (position of the fitted peak) and β (width of the peak at half height) for the 200 reflection, degree of crystallinity (CI) and size of the crystallites determined from application of the Scherrer equation to 200 reflection for the hemp fibers, raw and after the pretreatment step indicated in the first column.

Fiber Sample	200 (±0.05°)		CI (%)	Crystallite Size (Å)
	2θ_max_	β		
Raw hemp	22.41	2.18	74	39
First Alkali	22.71	1.62	79	55
Acid	22.57	1.60	80	55
Second Alkali	22.58	1.47	82	60
Enzymatic	22.66	1.45	80	61
Nanocellulose	22.58	1.45	80	61

## Data Availability

The research data generated in the current study are available in the open access Zenodo repository [41].

## Supplementary Materials

The following are available online, Figure S1: Comparison of the dynamic scanning calorimetry results obtained for the nanocellulose and the handsheet, Figure S2: Images of the surfaces of the handsheet obtained with a ZEISS LSM900 confocal microscope (Carl Zeiss Microscopy GmbH, Germany), Figure S3: Comparison of the weight and first derivative curves obtained from the thermogravimetric analysis of the nanocellulose and the handsheet; Figure S4: Results of the dynamic mechanical analysis performed on strips cut from the hemp handsheet; Figure S5: Stress-strain curves obtained in the tensile test performed on strips cut from the hemp handsheet.

## Sample Availability

Samples of the compounds are not available from the authors.
