# Supplementary material for: Valorization of Byproducts of Hemp Multipurpose Crop: Short Non-Aligned Bast Fibers as a Source of Nanocellulose
Source: Molecules. 2021 Aug 4;26(16):4723. doi: 10.3390/molecules26164723 (PMC8400396; doi:10.3390/molecules26164723)
Supplement: Supplementary file 1 [file molecules-26-04723-s001.zip › molecules-1313917-supplementary.pdf]

Supplementary information

Valorization of byproducts of hemp multipurpose crops: short non-aligned bast fibers as a source of nanocellulose

Sara Dalle Vacche[1,*], Vijayaletchumy Karunakaran[1,2], Alessia Patrucco[3], Marina Zoccola[3], Loreleï Douard[4], Silvia Ronchetti[1], Marta Gallo[1], Aigoul Schreier[5], Yves Leterrier[5], Julien Bras[4], Davide Beneventi[d], Roberta Bongiovanni[1]

1 Politecnico di Torino, Department of Applied Science and Technology, Corso Duca degli Abruzzi 24, 10129 Turin, Italy; vijayaletchumy13@s.unikl.edu.my (V.K.); silvia.ronchetti@polito.it (S.M. R.); marta.gallo@polito.it (M.G.); roberta.bongiovanni@polito.it (R.B.)
2 Universiti Kuala Lumpur, Malaysian Institute of Chemical and Bioengineering Technology (UniKL MICET), Lot 1988 Kawasan Perindustrian Bandar Vendor, Taboh Naning, Alor Gajah, Melaka 78000, Malaysia
3 Consiglio Nazionale delle Ricerche, Istituto di Sistemi e Tecnologie Industriali Intelligenti per il Manifatturiero Avanzato, 13900 Biella, Italy; alessia.patrucco@stiima.cnr.it (A.P.); marina.zoccola@stiima.cnr.it (M.Z.)
4 Université Grenoble Alpes, CNRS, Grenoble INP, LGP2, F-38000 Grenoble, France; Lorelei.Douard@lgp2.grenoble-inp.fr (L.D.); julien.bras@grenoble-inp.fr (J.B.); davide.beneventi@pagora.grenoble-inp.fr (D.B.)
5 Laboratory for Processing of Advanced Composites (LPAC), Ecole Polytechnique Fédérale de Lausanne (EPFL), CH-1015 Lausanne, Switzerland; aigoul.schreier@epfl.ch (A.S.); yves.leterrier@epfl.ch (Y.L.)

* Correspondence: sara.dallevacche@polito.it

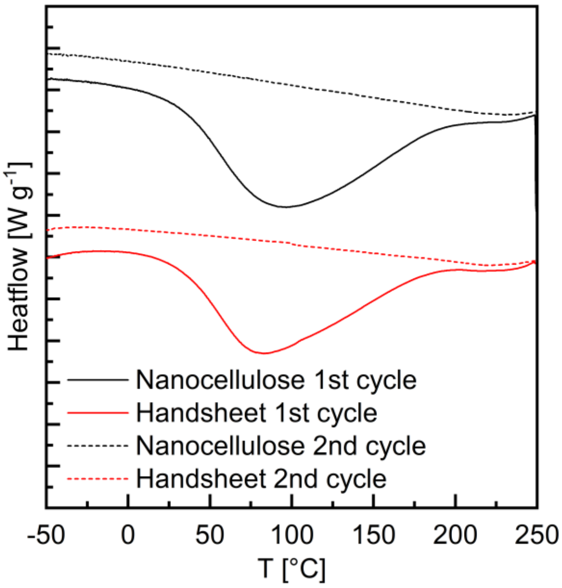

Figure S1. Comparison of the dynamic scanning calorimetry results obtained for the nanocellulose and the handsheet

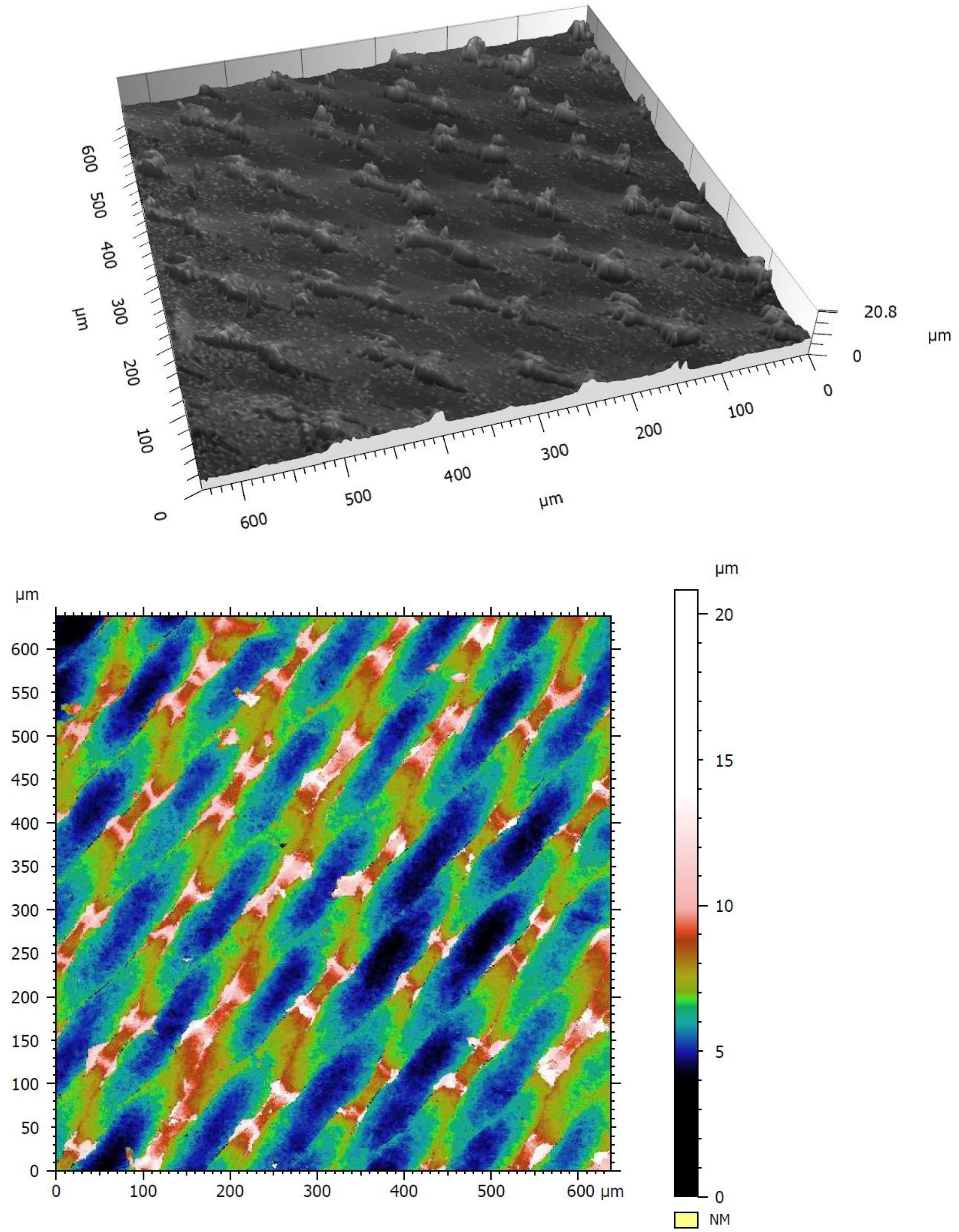

Figure S2. Images of the surfaces of the handsheet obtained with a ZEISS LSM900 confocal microscope (Carl Zeiss Microscopy GmbH, Germany)

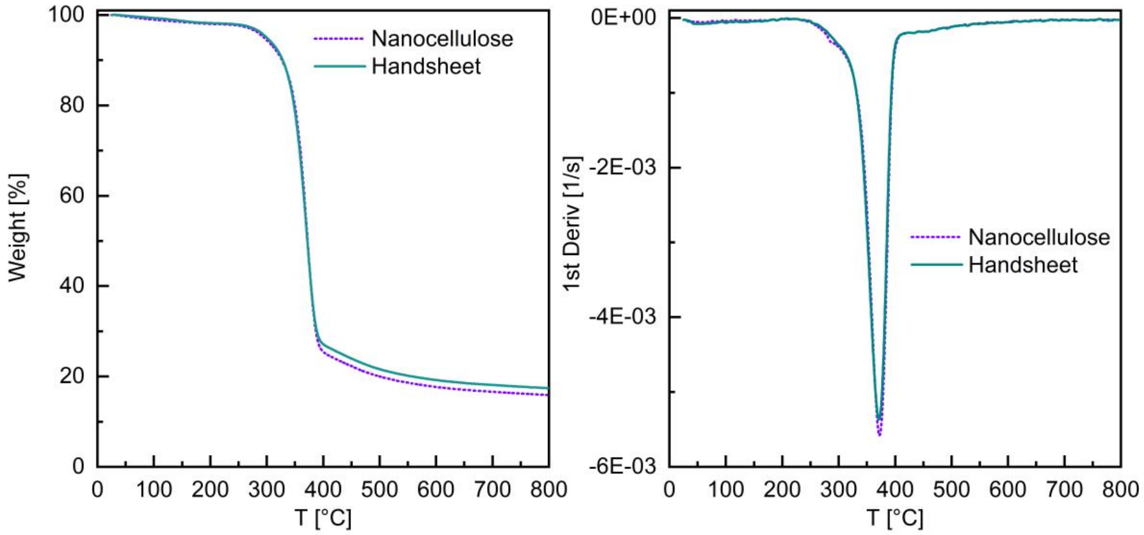

Figure S3. Comparison of the weight and first derivative curves obtained from the thermogravimetric analysis of the nanocellulose and the handsheet

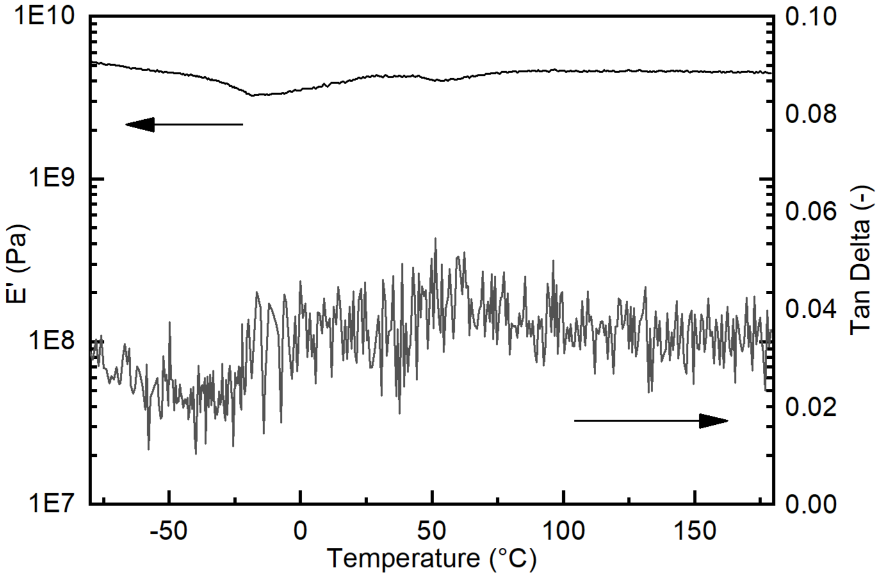

Figure S4. Results of the dynamic mechanical analysis performed on strips cut from the hemp handsheet

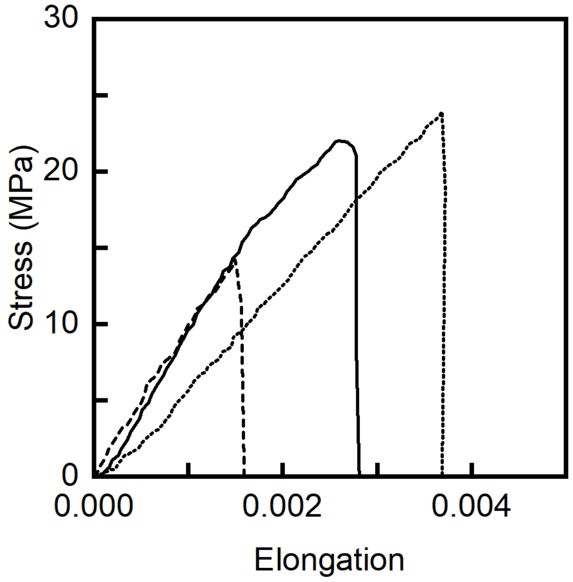

Figure S5. Stress-strain curves obtained in the tensile test performed on strips cut from the hemp handsheet
